# Resolution of cuffitis after removal surgical staple from ileal pouch-anal anastomosis

**DOI:** 10.1093/gastro/goaf002

**Published:** 2025-01-20

**Authors:** Bo Shen, Huaibin M Ko, Ravi Kiran, James Church

**Affiliations:** The Global Center for Integrated Colorectal Surgery and IBD Interventional Endoscopy, Center for Inflammatory Bowel Diseases, New York, NY, USA; Division of Anatomic Pathology, Columbia University Irving Medical Center/NewYork Presbyterian Hospital, New York, NY, USA; The Global Center for Integrated Colorectal Surgery and IBD Interventional Endoscopy, Center for Inflammatory Bowel Diseases, New York, NY, USA; The Global Center for Integrated Colorectal Surgery and IBD Interventional Endoscopy, Center for Inflammatory Bowel Diseases, New York, NY, USA

## Introduction

Cuffitis is a common phenotype of inflammatory disorder located at the rectal cuff of ileal pouch-anal anastomosis (IPAA) in those with underlying ulcerative colitis (UC) [1, 2]. Classic cuffitis is considered a form of remnant UC following IPAA without mucosectomy. Patients with cuffitis usually respond to topical mesalamine or topical corticosteroid therapy [3, 4]. Cuffitis can result from other etiologies, such as Crohn’s disease (CD) and prolapse, which often present with asymmetric distribution of the cuff inflammation [1, 5]. Common symptoms of cuffitis are urgency, frequency, bleeding, and pelvic pressure. Despite advances in the diagnosis and management of ileal pouch disorders, some patients with cuffitis poorly respond to topical and systemic medical therapy. In this brief report, we describe a case in which cuffitis was resolved by the removal of dislodged surgical staples from the anastomosis, suggesting a contributing role of the staples in cuffitis.

## Case report

A 44-year-old male was diagnosed as having proctosigmoid and then extensive UC in 2022 and was tried, but failed corticosteroids, infliximab, and adalimumab. He underwent subtotal colectomy, end ileostomy, and construction of a Hartmann pouch 3 months later. He developed a perianal fistula for which he had fistulotomy and responded. Four months later, he had a completion proctectomy, construction of a J Pouch, and diverting loop ileostomy with stoma closure 3 months afterward. Since the stoma closure, he has experienced urgency, frequency with bowel movements (10–12 times per day), nocturnal seepage, intermittent bleeding, and incomplete evacuation. Pouchoscopy was performed at a local doctor’s office, which showed ulcers at the vertical staple lines and anastomosis and was labeled as having CD of the pouch. He was started on oral antibiotics, adalimumab, and subsequent ustekinumab (on a 3-month stable dose). However, his symptoms persisted and came to the Pouch Center at Columbia University Irving Medical Center to get a second opinion.

**Figure 1.**
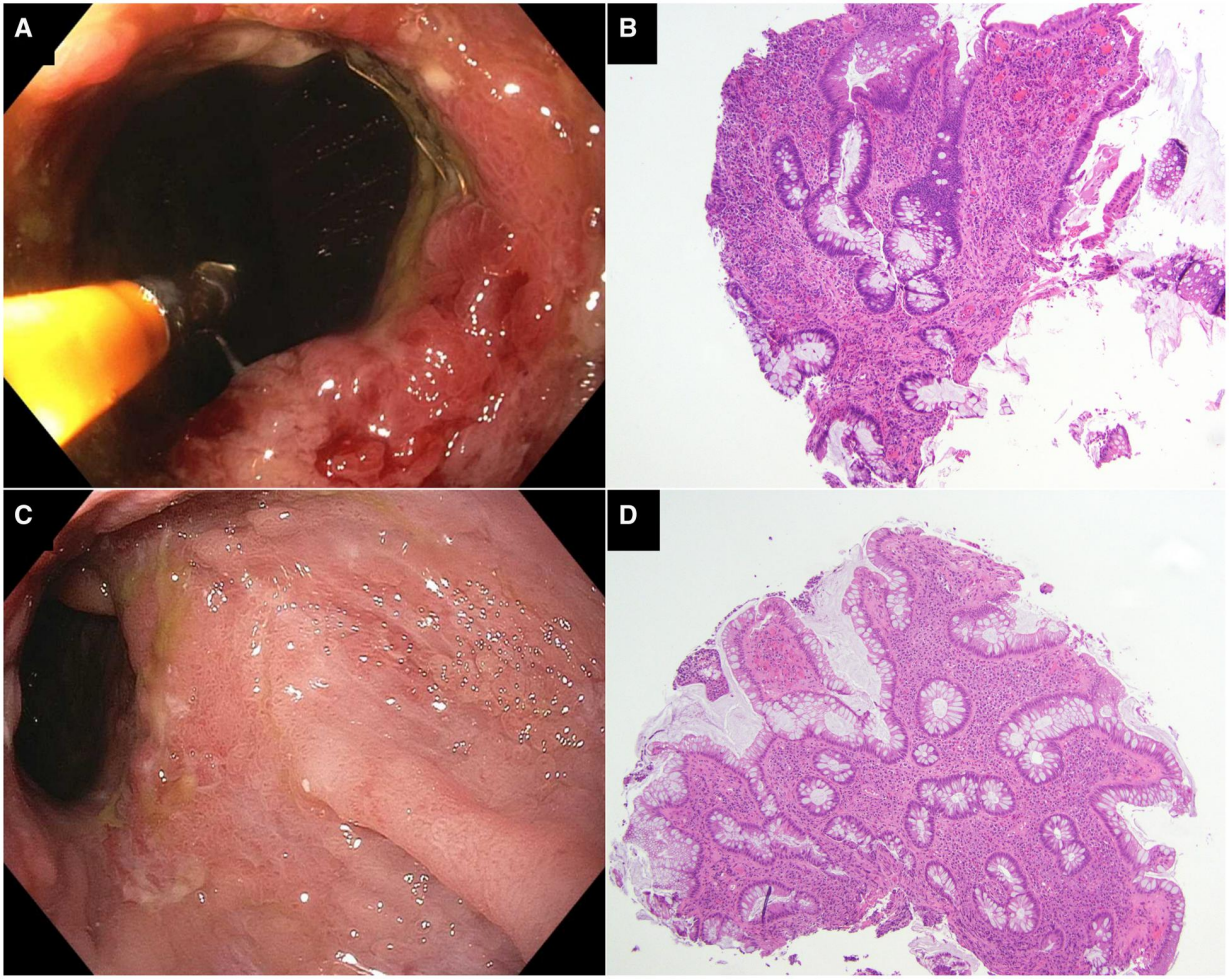
Refractory cuffitis before and after staple removal with pouchoscopy. (**A**) Severe circumferential “cobble-stoning” cuffitis with edema, erythema, nodularity, and friability. (**B**) Biopsy histology showed severe active inflammation with erosions and neutrophil infiltration. (**C**) Nearly complete resolution of cuff inflammation on pouchoscopy. (**D**) Residual chronic inflammation of the cuff on histology.

A repeat pouchoscopy was performed due to his refractory symptoms, which demonstrated a 4–5 cm circumferential cuffitis with nodularity, edema, and friability, and a normal pouch body and prepouch ileum. There were dislodged staples, two at the vertical staple line and five at the anastomosis, which were removed with biopsy forceps (Figure 1A). Histology of the biopsy showed severe active inflammation with erosions and predominant neutrophil infiltration (Figure 1B). The patient tolerated the procedure and was continued on ustekinumab.

The patient was followed up at the Pouch Center 2.5 months later with resolution of the symptoms, and bowel frequency was 5–8 times a day without incontinence, nocturnal seepage, or bleeding. Pouchoscopy showed resolution of cuffitis except for mild nodularity (Figure 1C), patent anastomosis, and normal pouch body and prepouch ileum. Additional six dislodged staples at the vertical staple line of the pouch body were removed. Histology of the cuff biopsy showed crypt architectural irregularity and chronic inflammation (Figure 1D). The patient was advised to have a yearly pouchoscopy for disease monitoring.

## Discussion

The current case illustrates the possible contribution of surgical staples in the development of refractory cuffitis. Stapled IPAA without mucosectomy is routinely performed in patients with UC undergoing restorative proctocolectomy for medically refractory UC. The remnant 2–2.5 cm-long cuff often has histologic with or without endoscopic inflammation. A circumferentially inflamed cuff has been considered a remnant UC in IPAA without mucosectomy [1]. However, circumferential cuffitis refractory to topical or systemic anti-inflammatory therapy, like in this patient, prompted the investigation of other contributing factors to cuffitis. Endoscopic removal of dislodged staples resulted in significant improvement of symptoms and endoscopic and histologic inflammation, suggesting that the surgical staples are contributing factors for cuff inflammation. In our clinical practice, we also noticed that the removal of dislodged staples helped the outcome of the endoscopic treatment of anastomotic strictures [6].

Most surgical staples are made from non-magnetic, titanium-based alloys that are magnetic resonance imaging-safe. Advantages of surgical staplers and staples listed by the Food and Drug Administration include quick placement, minimal tissue reaction, low risk of infection, and strong wound closure [7]. However, patients with inflammatory bowel disease undergoing resection, reconstruction, and anastomosis have underlying systemic and local inflammatory processes. The resection margin for CD has been defined based on macroscopic [8] vs microscopic [9] inflammation can impact the risk of postoperative disease recurrence, suggesting the presence of at least histologic inflammation at the margin. During the construction of IPAA, the margin of the rectal remnant stump is not free of inflammation. The interaction between the surgical staples and inflammatory tissues may lead to further inflammation, tissue ischemia, and even stricture or fistula formation. It is stipulated that the dislodged staples may introduce luminal microbiota in the anastomosis site, leading to inflammation and stricture formation. In transanal stapled procedures (including stapled hemorrhoidopexy, stapled transanal rectal resection, and transtar procedure), the removal of retained staples has been shown to improve patients’ symptoms [10]. It is not clear whether a hand-sewn anastomosis at IPAA can reduce the risk of inflammation. Endoscopically, the hand-sewn suture materials are hardly seen after IPAA becomes mature.

We believe that in some patients with underlying inflammatory bowel disease, surgical staples play a role in the development of local inflammation or stricture. It is advisable to remove dislodged staples at the anastomosis in those with ulcers, strictures, or nearby inflammation after more than 6 months after stoma closure.
